# Promising practices for the monitoring and evaluation of gender-based violence risk mitigation interventions in humanitarian response: a multi-methods study

**DOI:** 10.1186/s13031-022-00442-4

**Published:** 2022-03-05

**Authors:** Vandana Sharma, Emily Ausubel, Christine Heckman, Sonia Rastogi, Jocelyn T. D. Kelly

**Affiliations:** 1 Harvard T.H. Chan School of Public Health, 677 Huntington Avenue, Boston, MA 02115 USA; 2 Harvard Humanitarian Initiative, Cambridge, USA; 3 UNICEF, New York, USA; 4 Brigham and Women’s Hospital, Boston, USA

**Keywords:** Gender-based violence, Safety perceptions, Humanitarian contexts, GBV risk mitigation, Humanitarian response, Protection, Measurement, Monitoring and evaluation

## Abstract

**Background:**

Risks of gender-based violence (GBV) are exacerbated in humanitarian crises. GBV risk mitigation interventions aim to reduce exposure to GBV and ensure that humanitarian response actions and services themselves do not cause harm or increase the risk of violence. The 2015 IASC Guidelines for Integrating Gender-Based Violence Interventions in Humanitarian Action (‘GBV Guidelines’) are a globally endorsed resource that provides comprehensive guidance for all humanitarian actors and sectors on GBV risk mitigation. While uptake of GBV risk mitigation approaches across multiple humanitarian sectors has occurred, there is limited understanding of how to monitor and evaluate GBV risk mitigation interventions.

**Methods:**

A multi-methods study was conducted in 2019 to identify promising practices for the monitoring and evaluation (M&E) of GBV risk mitigation interventions in non-GBV sectors and to develop a set of illustrative case examples. The study included a comprehensive desk review of 145 articles, documents and resources from the published and grey literature, as well as 11 in-depth interviews and five focus group discussions with humanitarian practitioners. Using Dedoose software and a codebook developed a priori, qualitative data were transcribed and coded and a content analysis was conducted. Excerpts focusing on promising practices from the qualitative data and the desk review were analyzed together and grouped by thematic area. Similar promising practices were combined and consolidated to create a final list, and case examples were identified.

**Results:**

Current promising practices for M&E of GBV risk mitigation activities in the following categories are described: (1) Coordination and collaboration, (2) Designing M&E approaches and tools for GBV risk mitigation activities, (3) Contextualization, (4) Developing and selecting indicators, (5) Data collection, (6) Data analysis and use of findings, (7) Potential safety concerns for affected populations and staff, and (8) Staff capacity and engagement. These are supplemented with seven diverse case examples to illustrate application of the promising practices using real-world examples.

**Conclusion:**

This paper highlights current promising practices for M&E of GBV risk mitigation interventions in humanitarian response. Further application of these practices—alongside ongoing documentation of emerging approaches—will be critical to ensuring that GBV risk mitigation interventions are more rigorously tested with the aim of building the evidence base on the effectiveness of different GBV risk mitigation interventions within specific humanitarian sectors.

## Background

Gender-based violence (GBV) remains one of the most prevalent and life-threatening issues facing women and girls globally, as an estimated 30% of women experience some form of physical or sexual violence in their lifetime [1]. Evidence demonstrates that humanitarian crises—including conflict, natural disasters, and public health emergencies—exacerbate risks of GBV, as communities and families may face multiple destabilizing factors and affected populations may experience increased vulnerabilities which exacerbate pre-existing gender inequalities and create particular safety risks for women and girls [2–6]. Furthermore, with the humanitarian sector providing services to millions of displaced people worldwide in a variety of response areas, humanitarian actors have begun to recognize the ways in which the design and implementation of humanitarian programming across all sectors can also affect pre-existing gender inequalities and other GBV risks [7]. Humanitarian programs across all sectors must be designed to ensure they proactively identify and reduce the risks of violence related to humanitarian programming and minimize negative unintended consequences [8, 9]. This paper seeks to build the evidence base on the relationship between humanitarian programming and GBV risk, specifically focusing on the monitoring and evaluation of GBV risk mitigation interventions.

The foundation of the current humanitarian coordination system was established when the UN General Assembly created the Inter-Agency Standing Committee (IASC) in 1991. The IASC system brings together UN agencies, several international NGOs and a number of standing invitees to develop guidance and policies for a streamlined approach to humanitarian response worldwide. In 2005, the ‘humanitarian reform agenda' introduced the Cluster Approach, which is used for coordinating specific functional areas of operation in humanitarian emergencies. The current 11 clusters are Camp Coordination and Camp Management (CCCM), Early Recovery, Education, Emergency Telecommunications, Food Security, Health, Logistics, Nutrition, Protection, Shelter, and Water, Sanitation and Hygiene (WASH). The IASC’s definition of protection is “… all activities aimed at obtaining full respect for the rights of the individual in accordance with the letter and the spirit of the relevant bodies of law (i.e., International Human Rights Law (IHRL), International Humanitarian Law, International Refugee law (IRL))” [10]. The Protection Cluster is further broken down into four Areas of Responsibility: Gender-Based Violence, Child Protection, Land, Housing and Property and Mine Action.

In 2015, the IASC launched the Guidelines for Integrating Gender-Based Violence Interventions in Humanitarian Action (‘GBV Guidelines’), a revision to guidelines first released in 2005 [11]. These Guidelines serve as the first comprehensive interagency guidance that focus on GBV risk mitigation for all humanitarian actors and sectors. Within the GBV Guidelines, the IASC differentiates between GBV “prevention,” GBV “response” and GBV “risk mitigation.” GBV prevention is defined as actions that aim to prevent GBV from occurring in the first place, such as activities targeting underlying drivers of GBV and those that promote gender equality. GBV response refers to activities related to care and assistance for survivors of GBV, including medical, psychosocial and legal services. GBV risk mitigation is described as “reducing the risk of exposure to GBV (*e.g., ensuring that reports of ‘hot spots’ are immediately addressed through risk-reduction strategies; ensuring sufficient lighting and security patrols are in place from the onset of establishing displacement camps; *etc*.*)” [11].

An underlying principle of the GBV Guidelines is that GBV-related risks are affected by—and have implications for—all humanitarian sectors. As such, addressing GBV cannot be relegated to a single standalone GBV sector; on the contrary, it needs to be treated as a collective responsibility that cuts across the entire humanitarian system. The GBV Guidelines provide guidance for all of the sectors, clusters and areas of responsibility, aiming to help colleagues working in other sectors implement GBV risk mitigation activities even in the absence of specialized support. Specifically, they emphasize the essential actions that should be undertaken across other (non-GBV) sectors to protect those affected by crises from GBV risks across all stages of preparedness and response, from pre-emergency to recovery.

The GBV risk mitigation recommendations outlined in the GBV Guidelines seek to ensure that humanitarian actions and services do not cause harm and are responsive and sensitive to the diversity of needs, capacities and abilities among affected communities. As the GBV Guidelines have been rolled out, progress has been made across the humanitarian system to institutionalize GBV risk mitigation practices within agency policies, procedures and programming [12, 13]. However, there remains limited evidence on how, and to what extent, GBV risk mitigation activities improve GBV-related outcomes, such as women and girls’ safety. Practitioner insights and field reports suggest that GBV risk mitigation *does* lead to women reporting they feel safer, but a recent review reports that no rigorous impact evaluations have been completed to date to formally assess and document these outcomes [14]. In addition, there is limited evidence on how reducing GBV risk may contribute to key outcomes of interest for other humanitarian sectors (such as school enrollment and attendance (Education sector), malnutrition rates (Nutrition and Food Security sectors), and access to water and sanitation facilities (WASH sector) [15, 16]. These evidence gaps may be driven to some degree by the fact that there is limited understanding of how to measure the effectiveness of GBV risk mitigation actions—especially when the actions are implemented during acute crises, across a variety of sectors and, sometimes, by individuals who are less familiar with GBV-related data [16]. In particular, there are concerns around the safety and ethics of data collection and measurement of GBV risks by non-GBV specialists, and lack of clarity about which types of data would be appropriate for determining success of risk mitigation interventions [7, 11, 16].

As humanitarian practitioners across non-GBV sectors integrate GBV risk mitigation activities within their own programming, there is a need for guidance and promising practices for monitoring implementation and measuring GBV-related, as well as sector-specific, outcomes of these efforts. Incorporating appropriate measurement considerations in the design of these interventions will be critical to ensure that GBV risk mitigation activities are safely monitored, tested and evaluated and to identify which activities are particularly effective within each sector as well as to capture any potential unintended consequences. The aim of this multi-methods research study was to identify and develop a set of promising practices relevant to the measurement of GBV risk mitigation effectiveness across sectors that can guide future efforts within the humanitarian system.

## Methods

A multi-methods study was conducted from March to December 2019 by researchers at the Harvard Humanitarian Initiative (HHI) in partnership with UNICEF to identify promising practices for measurement of GBV risk mitigation activities in non-GBV sectors in humanitarian settings. The study included a systematic desk review, as well as qualitative in-depth interviews (IDIs) and focus group discussions (FGDs), to gather existing perspectives and documented practices from humanitarian professionals and researchers on the monitoring and evaluation (M&E) of GBV risk mitigation interventions in humanitarian settings with focus on measuring effectiveness.

1. Systematic mapping and in-depth desk review

### Study design

A systematic mapping and in-depth desk review of published, peer-reviewed journal articles, as well as grey literature, was undertaken between March and May 2019. Full details of the methodology are published elsewhere [14]. An explicit search strategy was used to identify relevant articles, documents, tools, reports, guidelines and case examples within a range of electronic databases and specialist websites. Key words were related to GBV, humanitarian response, GBV risk mitigation or safety, program utilization, measurement, assessment and evaluation. An open call for submission of relevant resources was also released and circulated among public health and humanitarian professional networks and communities.

The documents gathered from the database and website search and the call for submissions included project documents, guidance notes, and reports, as well as peer-reviewed academic journal articles. These documents focused on GBV risk mitigation interventions in humanitarian settings, as well as on methods for carrying out M&E of these interventions. In particular, the desk review focused on analyzing interventions for GBV risk mitigation carried out by non-GBV specialists in other humanitarian sectors.

### Screening and inclusion criteria

Overall, a total of 2108 documents were identified, including 1788 peer reviewed journal articles, 299 documents from the grey literature, and 21 resources submitted through the open call for materials. These documents were reviewed given the inclusion criteria, which included the following:

1. The document was published after 2005, when the IASC Guidelines for Gender-Based Violence Interventions in Humanitarians Settings were first published;
2. Documents were in the English language (though no language restrictions were placed on the open call for resources);
3. The document specifically discussed GBV risk mitigation activities, defined as “activities that reduce the exposure to GBV by addressing contributing factors,” as distinguished from GBV prevention and GBV response activities;
4. The document discussed GBV risk mitigation specifically in the context of humanitarian operations, including any phase of humanitarian response and all non-GBV sectors within the humanitarian cluster system;
5. The document discussed GBV risk mitigation activities that have incorporated measurement of outcomes or impact, both related to GBV and other sectors’ outcomes, including routine program monitoring as well as evaluations of GBV risk mitigation activities.

No restrictions were placed on the type of document, the geographic region, or the type of humanitarian crisis it discussed (acute versus protracted crises or natural disaster versus conflict).

Ultimately, through a title and abstract review of all 2,108 identified documents, 311 documents met the inclusion criteria for analysis, which was further reduced to 145 after a full text review. A total of 112 tools, such as safety audit instruments and qualitative interview guides were extracted from the final set of included documents. Analysis of the final set of documents involved extracting data from each document into a matrix with numerous categories to facilitate thematic analysis including: type of document, sector, country, theme, details of GBV risk mitigation activities and their monitoring and evaluation, tools, indicators, challenges and promising practices.

The detailed methodology and main findings of the systematic review are presented in a separate paper [14]. For the purposes of the current analysis, the extracted data that specifically addressed emerging promising practices related to the measurement of GBV risk mitigation activities were analyzed.

2. Qualitative data collection

### Study design and sampling

In addition to the desk review, 11 individual semi-structured IDIs (n = 11 participants) and five FGDs (n = 14 participants) were conducted between August and December 2019. Participants were recruited using purposive sampling in consultation with UNICEF, the interagency team involved in coordination and implementation of the GBV Guidelines and the study’s Technical Advisory Group. Participating key informants were also asked to recommend other humanitarian practitioners to consider for inclusion in the study. The formal inclusion criteria for participation in the study included: men and women above the age of 18 who were working in the humanitarian field in any sector (both GBV and non-GBV sectors) and had experience or technical expertise on the measurement of outcomes and/or evaluation of GBV risk mitigation activities during humanitarian response efforts. Staff members at NGOs, multilateral organizations or other types of organizations who were field practitioners or researchers were eligible. There were no predetermined geographic restrictions.

The final list of key informants included those chosen due to their roles as humanitarian practitioners and researchers, both in the area of GBV and in other sectors, including Food Security, Water, Sanitation and Hygiene (WASH), Nutrition, Health, Cash and Livelihoods. An emphasis was placed on identifying practitioners from a range of organizations, humanitarian sectors, countries and humanitarian settings. In some cases, key informants requested that additional colleagues or team members from their organizations participate in the interview together. In these cases, focus groups were convened rather than individual interviews.

### Data collection

The IDIs and FGDs were semi-structured, broadly following a discussion guide developed by the study authors. The guide was piloted and reviewed by the study’s Technical Advisory Group. The discussion guide included demographic questions (age, education level, length of time in the humanitarian sector, length of time working on GBV risk mitigation), as well as questions about the interviewee’s experience designing, implementing, monitoring and/or evaluating GBV risk mitigation interventions, including strategies, indicators, and tools. The discussion guide also included questions about promising practices for measuring the effectiveness of GBV risk mitigation activities. These questions included: (1) In your opinion, what are the best practices for measuring effectiveness and outcomes of GBV risk mitigation activities?, (2) What practices are you particularly proud of?, and (3) Follow up probes focused on the reasons for considering it a best practice, the countries, and sectors the best practice was used in, as well as specific lessons. Participants were not provided with a definition for “best practices” and thus shared practices based on their own interpretation of the term. Participants were also asked to share relevant tools and project documents for inclusion in the desk review. Interviews and FGDs were conducted in English remotely over audio recording software (Skype for Business 2016 and QuickTime Player 10.5), by members of the research team (EA, VS, JK). All interviews were conducted in private settings and audio recorded. IDIs and FGDs were between 40 and 100 min in duration.

### Ethical considerations

Verbal informed consent was obtained from each of the individuals interviewed. The consent process involved explaining the purpose of the interview, that participation in the interview was voluntary and the interview could be terminated at any point, any potential risks or discomforts that could arise as a result of the interview, and that all information collected would be kept confidential. All interviews were conducted in private. The research methodology—including the use of software programs for the interviews and audio recording—and the interview guides were approved by the Institutional Review Board (IRB) at Brigham and Women’s Hospital in Boston, Massachusetts.

### Qualitative data analysis

Interviews and FGDs were transcribed, and the transcripts were uploaded into Dedoose (Version 7.0.23, Los Angeles, California) qualitative analysis software system. A codebook, structured according to the discussion guide, was developed a priori (VS, JK, EA) for the main analysis and used to code transcripts. For the current analysis, a content analysis [17] was undertaken in which coded excerpts related to promising practices were extracted from all IDI and FGD transcripts and analyzed as described below. FGD and IDI data were not analyzed separately or differently as the goal was to extract and analyze all promising practices shared.

3. Analysis and synthesis of desk review and qualitative data

For this paper, the analysis focused specifically on the coded excerpts from the qualitative data that reported on promising practices, as well as the promising practices information extracted from the desk review matrix. These data were analyzed together to identify promising practices related to the measurement of GBV risk mitigation that have been documented or shared across any humanitarian sector, and a set of illustrative case examples. The research team decided to categorize the findings as “promising” practices rather than “best practices” due to the lack of rigorous analysis on this topic, the breadth of practices discussed in the literature and by the key informants, and the fact that this field continues to evolve as practitioners gain more experience in M&E for GBV risk mitigation.

The process involved compiling the identified promising practices into a matrix, along with the source (literature, IDI, FGD) and sector linked to each one. The promising practices were grouped together into thematic areas. Similar promising practices were combined and consolidated to create a final list. It should be noted that the research did not seek to prioritize or assess strength of each promising practice.

The final set of promising practices align with established recommendations for monitoring and evaluation in humanitarian contexts. In the event where a practice identified through the research was not supported by other sources or contradicted other recommendations, including those in the GBV Guidelines, the team considered each on a case-by-case basis, and consulted other sources of information. When there was insufficient evidence supporting a particular practice, or the team disagreed with a proposed practice, it was excluded. The promising practices were reviewed by the research team and members of the Technical Advisory Group.

Several relevant field examples were identified. Examples were selected to illustrate diverse promising practices and their application to a given context and sector. In several cases, key informants were requested to provide additional details and/or other information sources were identified to triangulate the available data and fill information gaps.

Consent was not obtained from interviewees to use direct quotes in the final report or article; therefore, direct quotes are not presented in the analysis. In place of direct quotes, views are summarized, which may, in some cases, provide less nuance.

## Results

### Document characteristics

In total, 145 documents were assessed as part of the systematic desk review. These documents span ten different humanitarian sectors listed in order from highest to lowest proportion of documents: protection, WASH, shelter, CCCM, livelihoods, health, education, food security, nutrition and mine action. Within these documents, 112 different tools for monitoring, evaluation and data collection were identified. The majority of tools were general and applicable across sectors. The next highest proportion of tools were specific to the WASH sector, followed by CCCM and Protection.

### Participant characteristics

Overall, 12 key informants were working at non-governmental organizations, while 11 worked at United Nations agencies, one worked jointly at a University research institution and a UN agency, and one worked at a donor agency at the time of the data collection (See Table 1). Regarding representation of humanitarian sectors, six worked in WASH, six worked in Nutrition, three worked in CCCM, three worked in Food Security, two worked in GBV and Protection, two worked in Livelihoods, and one worked in Health. Approximately three quarters of the interviewed key informants worked at the headquarters or regional level, while the other quarter worked in field positions. Many of those based at the headquarters or regional level had direct field experience and/or were providing ongoing direct technical support to country-level projects or a regional project with several countries.

**Table 1:** Participant demographic data

	Women N (%)	Men N (%)	All N (%)
Total	21	4	25
*Location*			
Field	5 (24%)	2 (50%)	7 (28%)
Headquarters/regional office	16 (76%)	2 (50%)	18 (72%)
*Type of organization*			
NGO	8 (38%)	4 (100%)	12 (48%)
UN Agency	12 (57%)	0	12 (48%)
Donor	1 (5%)	0	1 (4%)
*Years working in humanitarian sector*			
0–5 years	1 (5%)	2 (50%)	3 (12%)
5–10 years	6 (29%)	1 (25%)	7 (28%)
10 + years	10 (48%)	1 (25%)	11 (44%)
Unknown	4 (19%)	0	4 (16%)
*Geographic location*			
East Asia and Pacific	2 (10%)	0	2 (8%)
East and Southern Africa	4 (19%)	0	4 (16%)
Europe and Central Asia	8 (38%)	0	8 (32%)
Latin America and the Caribbean	0	0	0
Middle East and North Africa	0	0	0
North America	7 (33%)	2 (50%)	9 (36%)
South Asia	0	2 (50%)	2 (8%)
West and Central Africa	0	0	0

### Emerging promising practices

Emerging promising practices spanned 8 categories including: (1) Coordination and Collaboration, (2) Designing Monitoring and Evaluation Approaches and Tools for GBV Risk Mitigation Activities, (3) Contextualization, (4) Developing and Selecting Indicators, (5) Data Collection, (6) Data Analysis and Use of Findings, (7) Safety Concerns for Affected Populations and Staff, and (8) Staff Capacity and Engagement. The full list of promising practices identified as part of this research is provided in Table 2. The table presents the source of each promising practice (IDI/FGD versus desk review). To illustrate the use of the promising practices and highlight some of the lessons captured, case examples from programming and research are also provided (Tables 3, 4, 6, 7, 8, 9, 10).

**Table 2:**
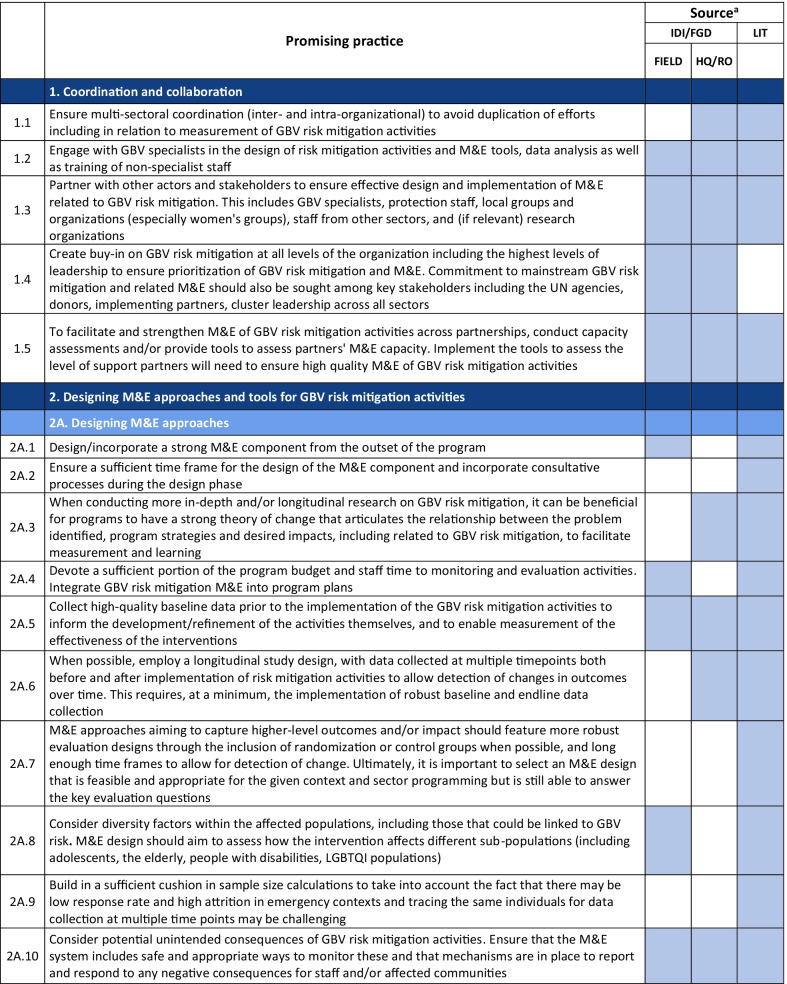

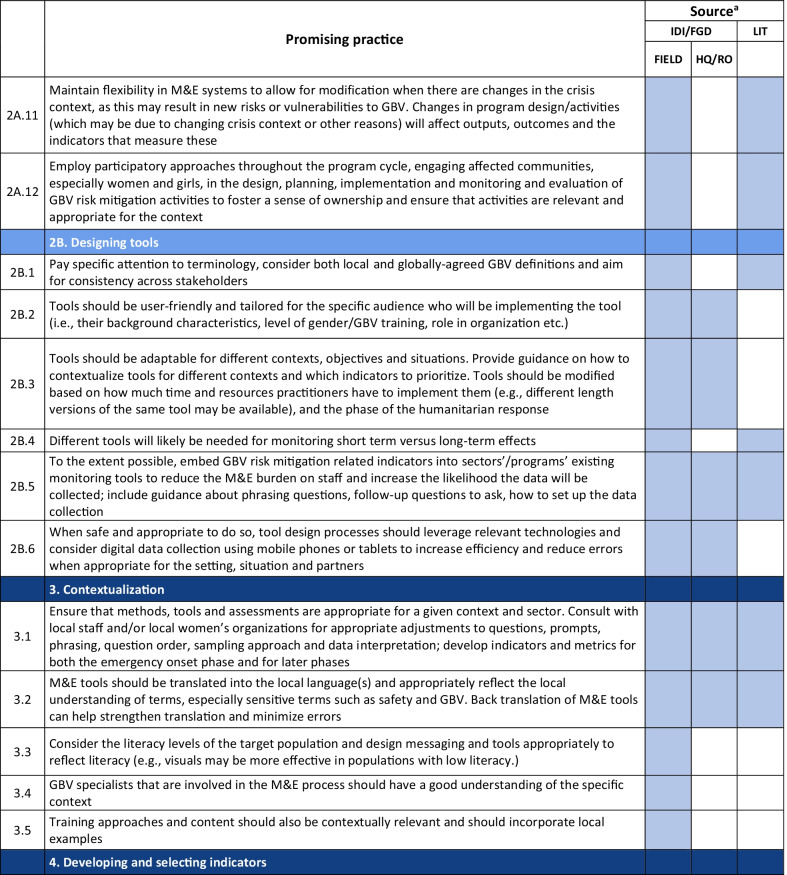

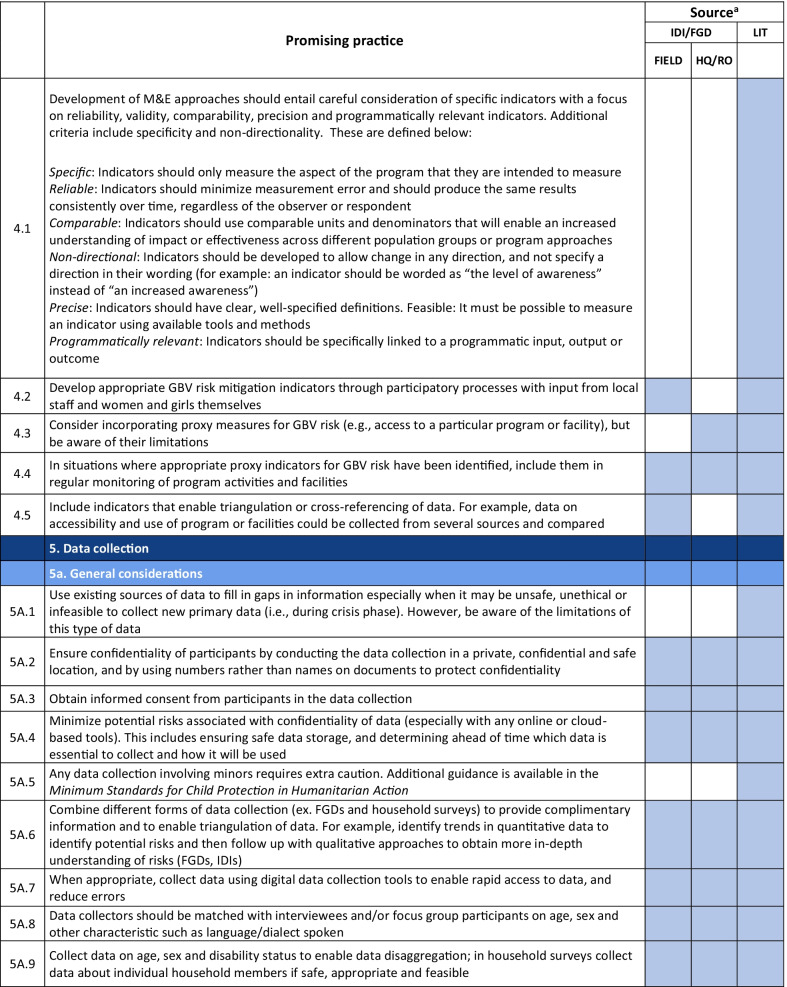

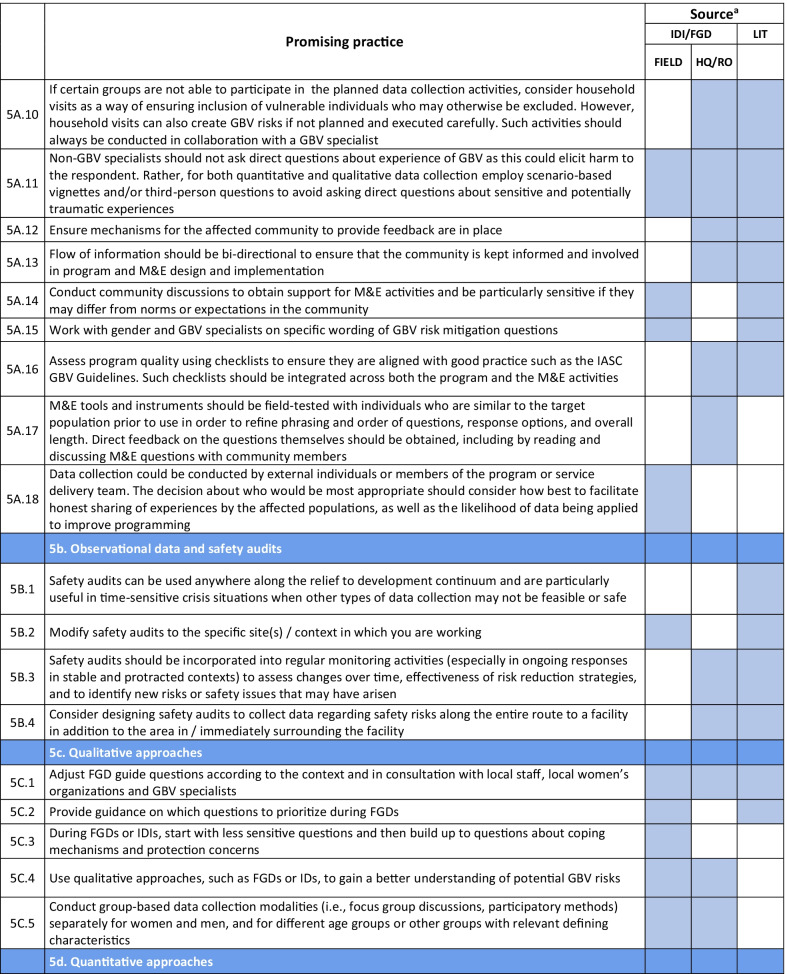

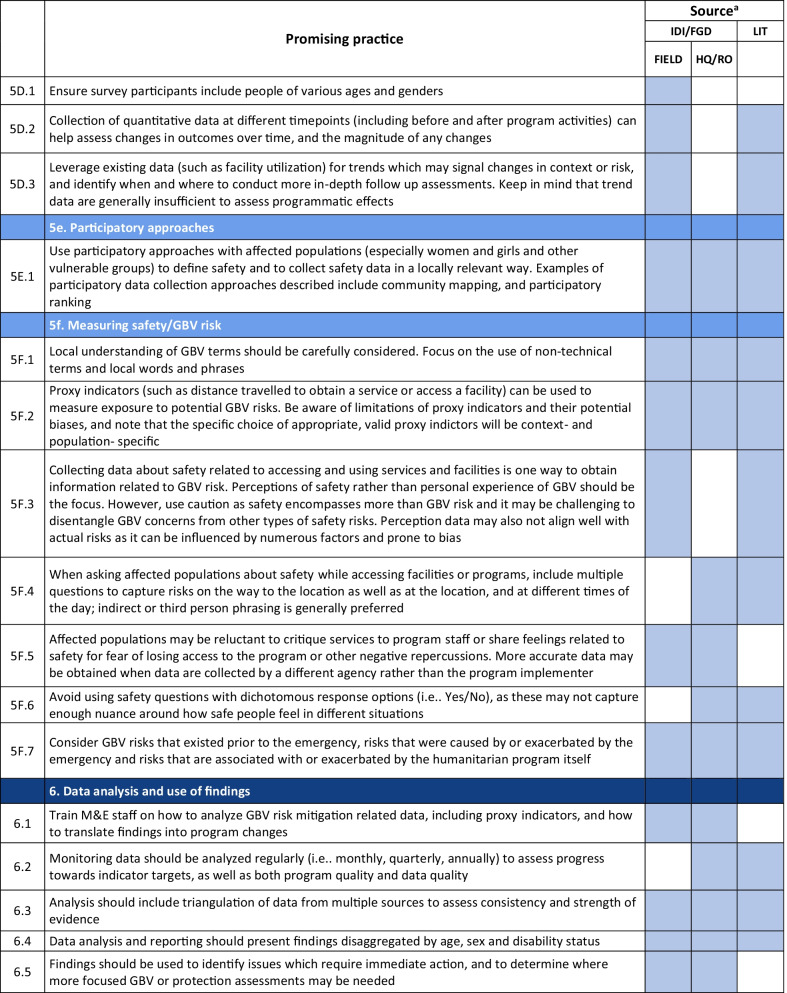

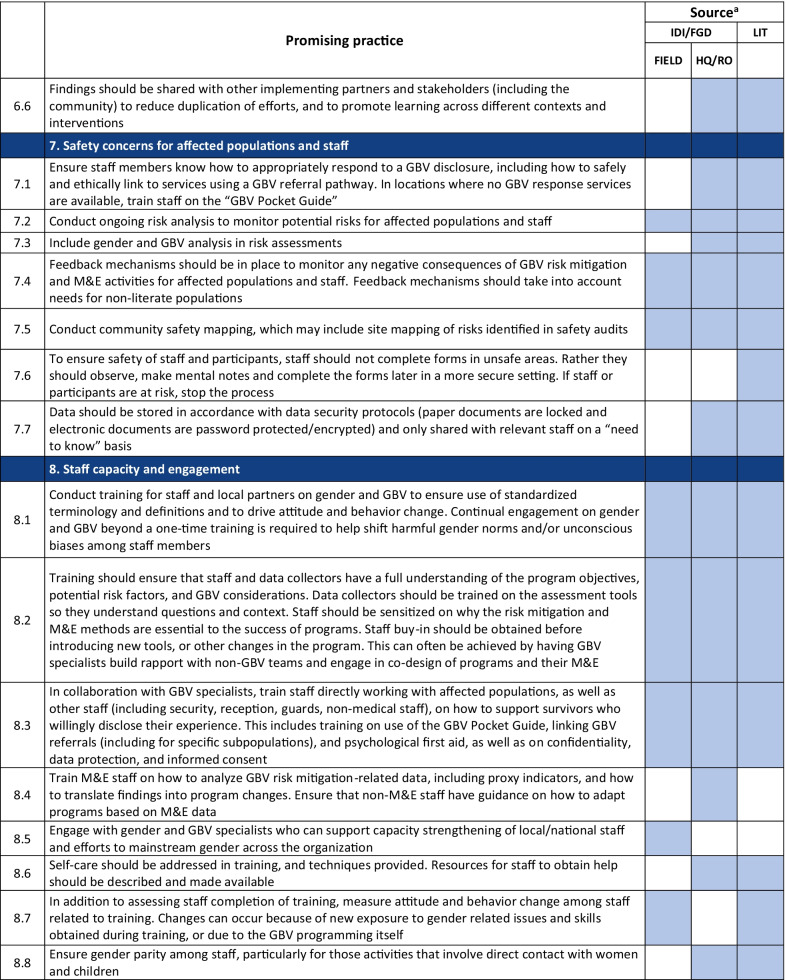
Full list of promising practices related to the monitoring and evaluation of GBV risk mitigation activities in humanitarian contexts

^a^This column indicates the source(s) of the recommendation—whether each promising practice was mentioned in one or more of the in-depth interviews or focus group discussions (IDI/FGD) or the literature (LIT) or both. IDI/FGD sources are also differentiated by the location type (field or headquarters/regional office (HQ/RO)) of the person or persons providing the promising practice

**Table 3:** Case example: participatory approaches to identifying risks and unintended consequences

*Countries*: Bangladesh, South Sudan *Sector*: WASH At the onset of the Rohingya response, WASH specialists worked quickly to construct latrines and bathing facilities for the refugees coming into Bangladesh from Myanmar. Safety audits by GBV actors, community feedback mechanisms, and a combination of focus group discussions and key informant interviews from both WASH and GBV actors were conducted to monitor GBV risk. As part of this dedicated effort to solicit feedback, humanitarian responders learned that many women were in fact not using these facilities. Based on this information, WASH, GBV, and camp management specialists jointly conducted new surveys, focus group discussions, and key informant interviews specifically with women and girls to better understand any concerns about WASH facilities. In one camp, this research uncovered a surprising unintended outcome of the WASH design: as a standard GBV risk-mitigation measure, WASH specialists had installed lamp posts near the latrines so that they were well-lit at night. However, women reported that this extra light helped make latrines more accessible to them at night, but also led boys and men to gather around the latrines to complete their homework or play cards in the evening. Overall, the placement of the lights resulted in women reporting they actually felt *less* safe in the area and ultimately avoided using these latrines Similar findings been reported in multiple humanitarian settings, including in Malakal camp in South Sudan, where lighting around latrines was identified as a crucial intervention by women and girls. Consultations with women and girls as part of the monitoring process helped identify some unintended consequences of the additional lighting in this setting. Men congregated near the well-lit latrines, which resulted in community leaders banning women from the facilities after dark which further limited their access to WASH facilities. Some women also voiced concern that lighting could potentially make vulnerable individuals more visible and an easier target [9]. While lighting around WASH facilities remains a basic intervention for reducing GBV risks, particularly in displacement camps or other camp-like setting with communal WASH facilities, these examples illustrate how placing lights *only* around WASH facilities—and nowhere else in the camp—can create the result in the opposite of the intended effect. Both IDIs as well as the literature highlighted the importance of using community-based approaches with direct engagement of women and girls to ensure that interventions are appropriate for the situation and context and to understand the actual effects of the interventions Humanitarian teams used the feedback in the Rohingya response to improve the lighting intervention and mitigate the GBV risks that were identified in several ways. Latrines and bathing areas were assigned to a cluster of houses so that community members did not have to walk as far and could coordinate with their neighbors and set schedules for using the facilities. Another proposed solution has been to provide personal solar lamps to women and girls to take with them when they access WASH facilities at night
*Key take aways*: Develop participatory monitoring mechanisms in collaboration with GBV specialists to identify GBV risks and allow women and girls to recommend solutions. Continue monitoring after the implementation of GBV risk mitigation actions to ensure the interventions are having the desired affects and to identify unintended outcomes, then feed learning back into programming. Engagement with women and girls should be ongoing throughout all phases of the project

**Table 4:** Case example: the importance of appropriate terminology and accurate translation

*Countries*: Bangladesh, Myanmar, DRC *Sector*: General During the Rohingya response, practitioners reported struggling to translate program information and interview questions into both Burmese and Rohingya, especially because Rohingya is not a written language. Determining direct translations for words like “gender” or “safety” was a challenge. As a result, one organization in Myanmar used a literal translation of “gender-based violence,” which translated as “sex-based violence.” This translation led to confusion among the organization’s staff, some of whom misinterpreted this to believe that physical violence was not a form of gender-based violence. A different organization used a longer translation that included all of the different types of gender-based violence for clarity. This definition seemed less efficient due to its length, but it yielded more accurate information Similar issues were described by a report on measuring gender-based violence in peacebuilding contexts [18]. When one organization tried to measure the level of female participation in communities in North and South Kivu provinces in eastern DRC, they struggled to translate between local languages and Swahili. Questions about women’s *participation* in community meetings were often understood to simply mean women’s *presence,* which did not fully capture the level of engagement that researchers were trying to measure. Taking these translation and interpretation issues into account, the organization expanded the indicator to include voicing opinions as well
*Key take aways*: Work with skilled translators and cultural mediators to translate M&E tools so that both local humanitarian staff and community members fully understand the terms and questions. Simpler terms may be more appropriate. Use back translation—a process where a different person translates the question that has been translated into the local language back into the original language—to understand where meanings may be lost or distorted. Always pilot questions with individuals representative of the affected populations before broadly implementing questions

1. Coordination and collaboration
  Considerations related to coordination of humanitarian activities were highlighted as a means to facilitate adoption of appropriate GBV risk mitigation and M&E of those activities. Promising practices addressed both coordination between different actors *within* organizations, as well as *between* organizations and sectors. Multisectoral coordination (both inter and intra-organizational) (1.1) was noted as critical in minimizing duplication of efforts, including related to M&E, and as an important approach to facilitate sharing of lessons and tools to support measurement across sectors. Working closely with GBV specialists (1.2) to design GBV risk mitigation activities and the accompanying M&E frameworks and tools, as well as to train staff on how to collect data about GBV risks without causing further harm to affected populations, was consistently noted. However, challenges of working with GBV specialists who were not familiar with specific humanitarian contexts or sector areas, as well as their limited availability, were also raised.
  Ensuring buy-in of GBV risk mitigation at all levels of the organization (1.4), especially at the highest levels of leadership was frequently described as critical for prioritization of GBV risk mitigation and its M&E. For countries in which the humanitarian cluster system had been activated, the importance of cluster-level commitment to GBV risk mitigation was also highlighted. Building partnerships with key actors and stakeholders (1.3), including local groups and organizations, was also described as an important approach to ensure effective design and implementation of M&E related to GBV risk mitigation. Key informants highlighted that local organizations and staff have the most direct knowledge about the current context and relevant cultural considerations. They also emphasized the benefits of having organizations or staff with more experience on M&E of GBV risk mitigation involved in staff and organizational capacity strengthening of those with less experience, especially given the sensitivity of the subject, challenges in measurement and the risk that any GBV-related activities have for causing further harm to affected populations. It was also cited that assessing partner capacity in M&E of GBV risk mitigation (1.5) and providing support accordingly is a helpful strategy. Key informants also described supportive supervision approaches during this process, such as accompanying recently trained staff on initial data collection visits.
2. Designing Monitoring and Evaluation Approaches and Tools for GBV Risk Mitigation Activities
  Promising practices related to M&E design have been grouped into two subcategories: A. Designing M&E approaches, and B. Designing tools.
  A. Designing M&E Approaches
    Several promising practices related to the design of M&E approaches were highlighted. While M&E for GBV risk mitigation has particular considerations (e.g., specific parameters around safe and ethical data collection, taking into account the availability of GBV response services, etc.), many of the practices cited are simply considered promising practice for humanitarian M&E in general. First, a strong M&E component should be developed from the outset of the program (2A.1), when GBV risk mitigation activities are being designed and budgeted. This ensures that planned M&E activities are well-integrated into the program, that they match the project design and objectives, and that data collection is feasible and timed appropriately. Similarly, contextualization is relevant across all thematic areas but is also further expanded in section 3 below.
    Designing a robust M&E approach requires several key components. A sufficient timeframe to design the M&E component (2A.2) is needed, and resource planning should ensure sufficient budget allocation for M&E activities (2A.4). For programs that seek to facilitate more intensive GBV risk mitigation measurement, it can be beneficial for GBV risk mitigation interventions to have a theory of change (2A.3) that articulates the relationship between the problem identified, program strategies, and desired impacts to facilitate measurement and learning, that can act as the roadmap for the M&E framework. A theory of change is particularly relevant for programs planning robust evaluations to assess effectiveness. While this an important goal which would facilitate M&E, it was recognized that having a theory of change is not a requirement for risk mitigation program implementation. The collection of high-quality baseline data (2A.5) prior to the implementation of GBV risk mitigation activities can help inform the development of GBV risk mitigation interventions and ensure they are tailored to the specific needs of the population. Baseline data is also critical to assess changes in key indicators over time, as well as the effectiveness of the GBV risk mitigation activities. As described in the indicator section below, the markers of success for GBV risk mitigation interventions do not include GBV prevalence or incidence for a number of reasons. There are significant ethical and safety considerations around data collection and management of this type of data, as well as the practical limitations of what such data can actually say about the effectiveness of a specific programmatic intervention.
    Given the limited evidence base on the effectiveness of GBV risk mitigation, more robust evaluation designs should be considered (2A.7) when it is deemed safe and appropriate and there is sufficient capacity, resources and time available to conduct such evaluations. It can be useful to incorporate longitudinal study designs when possible (2A.6), and other creative but robust designs can also be considered. Ultimately, the key is to consider the most appropriate M&E design for the given context and sector programming (2A.7).
    Diversity factors must be considered within the affected populations, including those factors that could be linked to GBV risk (2A.8). Interventions and associated M&E should be developed with relevant subgroups in mind (e.g., adolescent girls, the elderly, people living with disabilities, LGBTQI), as well as with an intersectional approach that recognizes power hierarchies and the intertwining and compounding discrimination, inequality and lived experiences of people with diverse co-existing identities (e.g., gender, ethnicity, disability status).
    In order to ensure GBV risk mitigation M&E effectively captures potential risks for vulnerable groups, it is important to be able to, at a minimum, disaggregate data by age, sex, and disability status. Disaggregation based on these characteristics can help identify differing levels of access and/or satisfaction with programming, as well as potential risk factors that are linked to GBV. Sample size calculations should take into consideration that there may be high attrition in emergency contexts (2A.9) and that it may be difficult to trace the same individuals for data collection at multiple time points.
    Potential unintended consequences (both negative and positive) of programmatic activities, including those implemented with the intention of reducing GBV risk, must also be considered as part of the design of risk mitigation programming and M&E systems. Examples of unintended consequences were raised across numerous sectors. For instance, cash-based initiatives have the potential to reinforce harmful gender norms if cash is provided primarily to men, or exacerbate household tensions if cash is provided primarily to women without an evidence-based social norm change component. Therefore, all cash programming should include consultations or other mechanisms to provide program participants an opportunity to define the scope of the intervention in a manner that is acceptable and appropriate. It is imperative that, as part of M&E systems, practitioners consider safe and appropriate ways of monitoring potential unintended consequences and that there are mechanisms in place where people can report any negative effects on staff and affected communities (2A.10). Practitioners must actively involve women and girls in the design and monitoring of activities to reduce the likelihood of the program or the M&E activities causing additional harm and to ensure the needs of women and girls are kept at the forefront of the intervention. See Table 3 for a case example on identifying unintended consequences using participatory approaches.
    In addition, M&E systems should retain some flexibility, as they may need to be modified if there are changes in the crisis context (2A.11) that result in new risks or vulnerabilities to GBV. In some cases, the program design or activities may also change, either due to changing crisis context or for other reasons. Such changes will also affect outputs, outcomes and the indicators that measure these, and thus require adjustments to M&E processes.
    Participatory methods were highly cited in the research as a way to engage the local community, especially women and girls, in the design, planning, implementation and M&E of GBV risk mitigation activities (2A.12). This approach was one of the most mentioned practices both within the literature and from the key informants. Participatory approaches should be integrated throughout the M&E system from indicator selection, to data collection to analysis. Participatory approaches foster collaboration between affected communities and humanitarian actors, and help to create knowledge that is owned and used by and for the stakeholders, and is relevant to the local context. In GBV risk mitigation M&E, participatory methods are essential and have been employed to engage different groups, such as women and girls, about their specific needs and safety concerns.
  B. Designing tools
    Several promising practices related to the design of tools and instruments emerged. First, given the wide variation in terminology used to describe forms of GBV and differences in local understandings, both local and globally-agreed GBV definitions should be considered and consistency across stakeholders in a given setting should be ensured (2B.1). The tools themselves should prioritize a user-friendly design to facilitate uptake and correct use (2B.2). Tools should include guidance about how and when to implement them, including specific details on phrasing of questions, follow-up questions or probes to ask, and how to implement FGDs or other data collection modalities in different circumstances. Tool guidance should be targeted to the background and capacities of the intended users. In addition, tools should be flexible enough for practitioners to modify them for different contexts, objectives and situations (2B.3).
    Furthermore, there may also be a need for different tools to measure short-term versus long-term effects of a program (2B.4). Guidance on how to contextualize tools for different settings and phases of an emergency and indicators to prioritize should be provided. For example, one key informant from the headquarters office of a donor agency commented on the differences between the first several weeks of an emergency compared to later phases and the potential benefit of developing different indicators for different phases of the emergency. Many respondents agreed that to the extent possible, GBV risk mitigation-related indicators should be embedded into various sectors’ and programs’ existing monitoring tools (2B.5) to reduce the burden on staff and increase the likelihood that the information will be collected and used to improve programming. Finally, tool design processes can also leverage relevant technology, as appropriate (2B.6). Specific technologies, such as KoBo Toolbox or smartphone apps, that allow rapid access to data by M&E teams for analysis, as well as easy and offline storage of data, were noted to be preferable given their increased efficiency and the reduction in errors. However, safety and ethical issues related to the use of technology should be carefully considered [19–21].
3. Contextualization
  Both the literature and key informants from numerous sectors underscored that both risk mitigation activities and the M&E of those activities should be adapted to the specific contexts of the humanitarian setting (3.1). These contexts may relate to the type of disaster, the geography of the area, local gender norms, other cultural considerations, and the overall political situation and level of coordination with the government. Local organizations, local staff and GBV specialists should be consulted to adapt tools, including on selecting target groups for interviews and surveys, phrasing of specific questions, question order, prompts and data interpretation.
  Several reflections from participants focused on translation and language considerations. These included ensuring M&E tools and accompanying materials are translated into the local languages, and appropriately reflect the local understanding of terms (3.2) [22, 23]. The literacy level of the target population should also be considered in the design of tools (3.3), and input should be sought from GBV specialists and experts to embed context-specific understanding (3.4). Trainings should also be adapted to the context and include local examples (3.5). Table 4 illustrates a case example focused on accurate translation.
4. Developing and Selecting Indicators
  Key informants and reviewed documents outlined factors to consider while developing and selecting indicators to assess within M&E systems. Many of these are applicable more broadly across humanitarian programming. For example, it was emphasized that development of M&E approaches should entail careful consideration of specific indicators with a focus on specificity, reliability, validity, comparability, precision, non-directionality and programmatically relevant indicators (4.1).
  It was emphasized that gender-sensitive GBV risk indicators should be developed through participatory processes with input from local staff and women and girls themselves (4.2). In alignment with globally endorsed ethical standards for GBV data collection, non-GBV specialists should not ask direct questions about experiences of GBV, as doing so could result in disclosure and potential re-traumatization or other forms of additional harm for survivors. Instead, proxy indicators should be leveraged to better understand GBV risk (4.3).
  Table 5 includes information about proxy indicators. For example, the number of women and girls accessing a program intervention, such as a food distribution or nutrition center, may serve as a proxy for GBV risk, as women and girls who are facing high GBV risk may not access services. Contextualized proxy indicators that have been vetted with GBV experts and local women and girls can help identify GBV risks when integrated into monitoring of program activities and facilities (4.4). However, the limitations to proxy indicators, including issues with biases and difficulty in interpretation, as well as potential sensitivities and safety considerations, should also be carefully considered. Furthermore, there is no clear consensus on reliable and valid proxy indicators for GBV risk.

**Table 5:** Proxy indicators

Proxy indicators are indirect measures of a result that are linked to the result by one or more assumptions [24]. They are often used when the most direct indicator is not practical or unsafe to measure
*Example*: Asking community members directly about experiences of GBV may place them at risk of harm. Proxy indicators that can provide safe information about GBV risk include:
Availability of services
Access to services
Perceptions of safety & exposure to GBV risk
It is important to be aware of limitations of proxy indicators and their potential biases, and note that the specific choice of appropriate, valid proxy indictors will be context-specific
  In addition, it is important to include indicators that enable triangulation or cross-referencing of data (4.5). For example, data on accessibility and use of program or facilities could be collected from several sources and compared. In the nutrition sector, teams could report the number of community members accessing nutrition interventions from routine data captured at nutrition facilities. In addition, community members themselves could be interviewed to assess barriers to access and level of service utilization. Confirming findings through multiple sources of information helps counter any potential biases that may exist and ensure findings are as accurate and reflective of the situation on the ground as possible.
5. Data collection
  Promising practices related to data collection have been grouped into several subcategories: A. General considerations applicable to all types of data collection, B. Observational methods and safety audits, C. Qualitative approaches, D. Quantitative approaches, E. Participatory approaches and F. Measuring GBV risk and safety.
  A. General considerations
    It is important to use existing sources of data to fill gaps in information, especially when it may be unsafe, unethical or infeasible to collect primary data (e.g., during the acute crisis phase) (5A.1). However, it is important to also note the limitations of existing data sources, as relevant.
    All data collection must follow existing ethical and safety guidelines [25–27] to ensure confidentiality (5A.2), that informed consent is obtained from all participants (5A.3), and that safe data storage practices are employed (5A.4) [28, 29]. Any data collection involving minors requires extra caution (5A.5). Additional guidance is available in the Minimum Standards for Child Protection in Humanitarian Action [30, 31].
    Data collection could be conducted by external individuals or by program staff, and the decision about who would be most appropriate should be carefully considered (5A.18). For example, program participants may be hesitant to provide honest feedback or concerns when speaking to program staff. Thus, data collection conducted by an external individual may improve the accuracy of the information provided. However, having program staff members collect the data may increase ownership and use of the data, and program staff have gained trust within the community in a way that an external individual may not have. Staff should also seek support from GBV specialists as needed.
    Data collectors should be matched with interviewees and/or focus group participants on age, sex and other characteristics (5A.8), and data on these characteristics should be collected and reported (5A.9). Data collector characteristics, such as ethnicity, language and religion, may influence reporting due to local inter-group dynamics and should be carefully considered. Data collectors should receive appropriate training and conduct the interviews or focus group discussions in the local language.
    Barriers to participation in data collection activities should be identified and reduced in order to ensure a diverse group of participants. This could include deliberate measures to reach women and girls in remote locations, individuals with disabilities and other under-represented groups (5A.10). Truly inclusive participation may require visiting individuals in their homes and carrying out data collection at different times of the day. However, assessments/monitoring involving home visits may create additional risks and thus should always be planned with GBV specialists.
    GBV risk mitigation-related data collection should not include asking direct questions regarding community or individuals’ personal experiences of GBV, as this could elicit harm to the respondent (5A.11). This was one of the most frequently repeated points, both in the IDIs/FGDs and throughout the literature. Using scenario-based vignettes or third-person questions can help obtain information about GBV risk in the community in a more general way. For example, questions could ask whether the respondent has heard of any safety concerns related to a specific program, or about how safe or unsafe a person similar in age and sex might feel in accessing a particular program or facility. Questions should be designed with the help of a GBV specialist (5A.15), and practitioners should leverage existing, recommended tools. It is also a promising practice to start with less sensitive questions and build rapport before engaging in questions that might be more sensitive, such as those that involve safety concerns and coping mechanisms (5C.3).
    Several promising practices related to community engagement emerged. First, mechanisms for the affected community to provide feedback must be in place (5A.12) to identify unintended consequences, potential harms and other concerns of the affected population. However, the flow of information should not be just unidirectional. Instead, the community should be kept informed of and involved in M&E design and implementation (5A.13). Furthermore, community dialogue on planned M&E activities should be undertaken to obtain buy-in, especially when activities may differ from community norms and expectations (5A.14).
    It is important to leverage existing resources, including the IASC GBV Guidelines (5A.16), which includes a section on M&E for the mitigation of GBV across all sectors of humanitarian response [10]. Staff should have access to the GBV Guidelines and use the GBV Pocket Guide (available in booklet form and as a mobile application) to help prepare frontline workers, including data collectors, to respond appropriately in case someone shares an experience of GBV with them [32, 33]. Checklists can be incorporated into program monitoring activities to track progress on how well GBV risk mitigation and gender considerations have been integrated into programming and whether the programming meets established or recommended standards, such as those within the IASC GBV Guidelines (5A.16).
    M&E tools should be field-tested with individuals similar to the target population to refine phrasing and ordering of questions, response options, and overall length. Direct feedback on the questions should be obtained, including by reading and discussing M&E questions with community members (5A.17). The use of digital data collection tools to enable rapid access to data and reduce errors (5A.7) was also described as a promising practice. Digital tools can be beneficial in the right context, but may not be appropriate or feasible for every setting or situation.
  B. Observational data and Safety Audits
    Observational data collected via safety audits and other tools is an important modality used in numerous sectors, including WASH, shelter, and CCCM. A safety audit uses observation and sometimes consultation with affected communities, especially women and girls, to assess sector-specific GBV risks related to the environment, resource availability and provision of humanitarian services and assistance [34]. Several promising practices apply to this type of data. First, safety audits can be implemented anywhere along the humanitarian to development continuum and are particularly useful in time-sensitive crisis situations when the collection of other quantitative or qualitative data may not be feasible or safe (5B.1). The content and timing of safety audits should be adapted for different sectors, types of facilities and for specific contexts (5B.2).
    If appropriate for the program, safety audits can be incorporated into routine monitoring activities and conducted regularly to measure change in safety and risk over time (5B.3). When conducting safety audits for specific settings, it is important that staff consider the security context of the surrounding areas and possible safety risks that may arise in accessing the environment, facility or site (5B.4), such as the route to and from a facility. Depending on the context, participatory methods, such as community safety walks or community safety mapping, can also be incorporated into safety audits.
  C. Qualitative Approaches
    Focus group discussions and/or individual interviews are particularly useful to gain a better understanding of potential GBV risks (5C.4). With respect to FGDs, a number of promising practices emerged. It is advisable for practitioners to consult with a GBV specialist to develop FGD questions related to GBV risk mitigation (5C.1). Prioritization of questions should be based on program and sector needs, the program’s theory of change (if relevant), and context, and guidance on how to prioritize questions should be provided (5C.2). Questions should be adapted to the local context and translated appropriately, and staff must be adequately trained. Group composition is an important consideration, and groups should be divided by demographics characteristics such as age and sex (5C.5), and potentially other qualities depending on the context. For example, in some contexts it may be necessary to make adjustments in how focus groups are conducted based on dynamics between displaced populations and host communities, power dynamics linked to marital status and other considerations.
  D. Quantitative Approaches
    As described above, ensuring the collection of sex, age and disability status data and the disaggregation of data by these factors (5A.9) is important. This was particularly emphasized in relation to quantitative data collection to better understand how GBV risks and risk mitigation interventions affect different sub-groups within the target population. Ensuring survey participants include people of various ages and genders (5D.1) and collecting individual-level data for household members during household surveys were also mentioned. One key informant in the field office of an NGO addressed this point directly, explaining the limitation of teams carrying out household surveys without systematically thinking about including both men and women. For example, deciding only to talk with heads of households, who are disproportionately men, leads to a missed opportunity to collect data that could potentially be used to inform GBV risk mitigation actions. However, it is important to note that it may not always be safe, appropriate or feasible to collect individual-level data during household surveys.
    Assessing the effectiveness of GBV risk mitigation activities was not frequently mentioned during the interviews. However, it was noted that quantitative data collected at different time points (for example pre- and post-intervention) allows assessment of changes in key indicators over time (5D.2) and in some cases the effectiveness of the interventions. Table 6 provides a case example on measuring change related to GBV risk mitigation activities. In addition, assessing existing data, including facility utilization data, or analysis of GBV patterns and trends provided by GBV specialists, was noted to provide important insights into broader GBV patterns and signal potential changes in context or risk (5D.3). For example, practitioners can use trend data to better understand what demographic groups should be included in assessments to identify GBV risks. It is also worth noting that often the most valuable information on GBV patterns and trends is gathered through qualitative methodologies (supervised by GBV specialists). As described earlier, data collection, sharing and analysis for GBV risk mitigation purposes should never include the actual number of GBV incidents, due to safety and ethical considerations and limited utility from an M&E standpoint. See Table 7 for a case example on using administrative data to identify trends in program/facility use. It is important to note that administrative trend data may provide little information on short-term or more subtle changes for a specific indicator or the needs of specific subgroups within the population. Such trend data may be insufficient to assess programmatic effects, since changes in trend data may also reflect changes in the overall context that are unrelated to program activities. Caution must be employed when interpreting such data.

**Table 6:** Case example: measuring change in GBV-related outcomes-assessment of cookstove and fuel projects and GBV risk

*Countries*: Global, Kenya, Sudan, DRC *Sector*: Food security, Livelihoods A systematic review conducted in 2016 [35] of the evidence on cookstove and fuel programs in humanitarian settings found that 15 out of the 126 projects reviewed (12%) included at least one objective related to GBV. The review emphasized that further evidence is needed to demonstrate the impact of cookstove and fuel interventions on GBV outcomes *Impact on indicators related to exposure to GBV risk*: Of the 15 projects reviewed that had at least one GBV objective, four measured baseline factors related to risk of GBV during firewood collection, but only two of the projects measured the same outcomes at endline (the Berkeley Darfur Stove Project in Darfur, Sudan [36], and a cookstove project in the Democratic Republic of the Congo (DRC) implemented by the Women’s Refugee Commission [37]). Both of the projects assessed proxy measures related to GBV risk exposure including: number of firewood collection trips, number of hours spent during one round trip to collect firewood, and number of kilometers traveled during one round trip to collect firewood. Both projects reported changes in these indicators with implementation of the cookstove intervention. For example, in DRC, the average number of hours women spent per firewood collection trip dropped from 6 to 4 h, and the number of trips dropped by half
*Key take away*: It can be strategic for humanitarian agencies and practitioners to begin to measure GBV-related indicators at baseline and endline in order to assess the effectiveness of GBV risk mitigation activities and any potential unintended consequences. Non-GBV practitioners should NOT assess incidence of GBV. It can be helpful to include proxy indicators that—through other data points, such as distance traveled—indirectly measure exposure to GBV risk. However, the design and use of such indicators should always be done in collaboration with a GBV specialist and enumerators must be trained in how to provide referrals to GBV response services. More robust evaluations of GBV risk mitigation activities should be undertaken together with GBV specialists when other necessary conditions required to ensure safety of participants are in place. These more rigorous evaluations are critically needed to build the evidence base on the effectiveness of GBV risk mitigation activities

**Table 7:** Case example: leveraging existing or routinely collected quantitative data to identify trends and potential risks

*Country*: Myanmar *Sector*: Health In camps in Rakhine state, Myanmar, health sector professionals collected routine monitoring data on the number of community members who were accessing each of the health centers in the camps. These data were disaggregated by sex and also by which camp the community members were coming from. Analysis of this data over time showed a decrease in the number of women accessing the health centers from certain camps and even from specific neighborhoods. This trend raised a red flag among humanitarian staff, as it was indicative of a potential change in GBV risk. For example, women in those particular locations may have stopped using the health centers because of increased in GBV-related safety concerns on the way to the health centers. In order to better understand the situation, consultations with women and girls were carried out to further investigate why women were not accessing health services. Women reported that they had to pass through new checkpoints to reach the health facilities, and that they were experiencing harassment at these checkpoints. The team used this information to improve their programming, adding accompanied transport services for women, children, and people with disabilities so that they did not have to walk through the checkpoint on their own. This intervention quickly increased access to health center services
*Key take away*: Leverage existing data or routinely collected monitoring data to identify changes in trends (such as decreases in access to services) that may signal potential red flags for GBV risk, and follow up with further qualitative assessments to better understand what factors have contributed to the change
  E. Participatory Approaches
    Participatory approaches should be considered as part of data collection on GBV risk mitigation (5E.1). Such participatory methods may include participatory safety rankings, or community / safety mapping activities in which specific groups are invited to draw maps of the site and indicate any places that they feel safe or unsafe. Some documents also suggest participatory safety walks, where community members accompany program staff on observational walks through the sites and point out any safety concerns they have. Participatory approaches focused on GBV risk mitigation should be planned and implemented in collaboration with GBV specialists and local staff.
  F. Measuring Safety / GBV Risk
    Given that GBV risk mitigation is an emerging area of programming and research, there is limited evidence about how non-GBV specialists can best measure GBV risk and safety. However, several promising practices have emerged from the review. First it is critical to understand and respect local understanding of GBV-related concepts (5F.1). In some languages, words such as gender and safety may not have clear or direct translations, and in certain contexts, talking explicitly about GBV or safety may lead to backlash from community members, or discomfort among program participants. Using terms that are more familiar to and accepted by the community can help avoid these issues. Working together with the community is key in ensuring the right terms are used in a culturally sensitive way.
    Proxy indicators (such as distance travelled to obtain a service or access a facility) can be used to measure exposure to potential GBV risks (5F.2). Proxy indicators may be particularly useful when local terms for a particular concept do not exist, or when asking questions related to GBV risk may not be feasible or safe. Community members, especially women and girls, can assist with identifying and/or adapting proxy indicators related to safety and access that correspond most closely with GBV risk. Local women’s organizations can also help to determine which proxies are appropriate within a given context. This process should always be undertaken in collaboration with a GBV specialist. It is also important to be aware of the limitations of proxy indicators and their potential biases and note that the specific choice of appropriate, valid proxy indictors will be context- and population-specific. One strategy to help address some of these limitations is to incorporate multiple proxy measures, rather than relying on one or two. Table 8 includes a case example on using proxy indicators to assess GBV risk.

**Table 8:** Case example: developing and testing proxy indicators for GBV risk exposure using participatory approaches

*Country*: Kenya *Sector*: Food security, Livelihoods The World Food Program (WFP) implemented the SAFE project in Kakuma and Dadaab refugee camps in Kenya with the aim of reducing exposure to the risk of GBV during firewood collection. The program included distributing fuel-efficient stoves, training on the effective use and maintenance of fuel-efficient stoves and sensitization on GBV to both the refugee and host community. As part of the program the team undertook a robust mixed methods evaluation in Kakuma with baseline and endline household data collection as well as focus group discussions to assess the effectiveness of the program [38]. The non-randomized design included a control group that did not participate in the program, a group that received the cookstoves and training on their use only, and a third group that received the cookstoves, the training on cookstove use, as well as the GBV sensitization To evaluate exposure to GBV risk the research team selected three proxy indicators: Time spent away from home to collect firewood Frequency of firewood collection Distance travelled to collect firewood Working with the refugees to obtain feedback on these measures, it became clear that, from their perspective, time spent away from home and distance traveled were not considered to be key factors related to exposure of GBV. Because of the great risks of violence within the host community, refugees indicated that they mostly collected wood in and around the camp and that these locations were dangerous. The most dangerous areas were reported to be along or near a riverbed which passes through the camp and surrounds the camp borders. Thus, GBV risk was not correlated with how far women traveled. Instead, it was determined that frequency of firewood collection was a better proxy to evaluate exposure to GBV risk among this population On the other hand, among the host community, all three proxy indicators were deemed to be relevant measures of GBV risk, as women and girls from the host population were traveling further to collect firewood than the refugee women and girls The study found that the intervention led to a reduction in the consumption of cooking fuels due the energy efficient stoves, and that led to a decrease in the frequency of firewood trips in both the refugee and host community, thereby reducing exposure to GBV
*Key take aways*: Proxy indicators for GBV risk can be a valuable way to measure exposure to potential GBV risks, and to assess impacts of GBV risk mitigation activities. However, it is important to be aware of limitations of proxy indicators and their potential biases, and note that the specific choice of appropriate, valid proxy indictors will be context- and population- specific. They must be developed in collaboration with a GBV specialist and with input from the target population. They must also take into account the specific context in order to ensure validity and reliability
    Both key informants and the reviewed documents emphasize that GBV specialists and non-GBV specialists can work together to craft questions about women and girls’ *perceptions* of safety rather than their personal *experiences* of GBV (5F.3). Collecting data about safety related to access and use of services and facilities can be useful to obtain GBV risk information while reducing the likelihood of spontaneous disclosure of sensitive personal experiences. However, safety encompasses more than GBV risk, and it may be challenging to disentangle GBV concerns from other types of safety risks. Perception data may also not align well with actual risks, as it can be influenced by numerous factors and be prone to bias. As an example, a respondent may not feel safe accessing a latrine because of the physical integrity of the structure, rather than due to GBV-related concerns. Affected populations may also not share their concerns or critique programs for fear of losing access to them. Therefore, more accurate data may be obtained when data are collected by a different agency rather than the program implementer (5F.5). Given these factors, measuring perceptions of safety has the potential for both under- and over-estimating GBV risk. Additional work is needed to develop technical guidance and sample questions and indicators on this topic.
    Because of these challenges, when asking affected populations about safety accessing services, use a number of techniques, data sources and data points to triangulate information (5F.4). This may include capturing risks while traveling to a facility, at the facility itself and at different times of the day, and use of indirect or third person phrasing so respondents do not disclose their own experiences. Pilot testing with local staff and affected populations, including women and girls, is essential. Table 9 includes a case example on triangulation.

**Table 9:** Case example: triangulating data to ensure validity of safety measurements

*Countries*: South Sudan and Ecuador *Sector*: Nutrition, Food Security and CCCM *South Sudan*: In South Sudan, a survey asked whether community members were able to access services without any safety concerns. In response, 98% of women reported not having any safety issues while accessing food distribution and nutrition services. However, during the period in question, one of the service sites was closed for several months due to nearby conflict. Therefore, contrary to the community member responses, the available service data suggested that there likely *were* safety concerns related to accessing the food and nutrition sites. Several factors may have contributed to these seemingly contradictory data points. It was possible that women stated they did not have safety concerns accessing services, simply because they were not accessing services at all. Alternatively, women may have been responding in a way that was perceived as favorable or acceptable to humanitarian responders (also called social desirability bias). It is also possible they simply interpreted the question differently than the survey had intended. Last, they may in fact have had no safety concerns, despite nearby conflict This example illustrates why it is critical to triangulate data from multiple sources to assess consistency and strength of findings and to “gut-check” whether data seem like they are measuring what they are intended to measure. The nutrition facility data in this case provided an independent source of information about access and demonstrated that the perception data may not be valid. This also highlights the benefit of cross-sector collaboration and sharing of information. One additional and important point relates to the definition of safety used and the need to be able to capture the right data to separate perceptions of personal safety and safety related to GBV risk from general insecurity and conflict-related concerns. In this case example, a broader definition of safety may have been used making it difficult to understand GBV-related risks *Ecuador*: During both baseline and endline assessments for a program in Ecuador, camp management specialists surveyed women about how safe they felt and what they thought could help improve their situation and access to services. Somewhat surprisingly, analysis of the data showed that, in the endline assessment, more women reported feeling unsafe than they had in the baseline assessment. However, by speaking with women further, the camp management specialists realized that more women had reported feeling unsafe at the endline, because they felt that something might actually be done about their concerns, whereas at the start of the program, they had assumed it was futile to report safety issues in the first place. This type of reporting effect, wherein an intervention leads to increased reporting of GBV risk, has also been reported in other projects and sites. Higher levels of reported risk are not always a “bad sign”, and may actually indicate increased disclosure of risk and/or trust in staff and program responsiveness
*Key takeaways*: Safety perceptions are by definition subjective and prone to biases, including changes in reporting patterns. This type of data must be triangulated with other data sources, such as situational analyses, observational studies or monitoring data to assess consistency and strength of the findings. Particular attention must be given to definitions, indicators and questions to ensure perceptions of personal safety and safety related to GBV risks are captured rather than only general insecurity and conflict-related concerns
    Other field experience data suggest that for quantitative indicators of safety, questions with dichotomous response options such as yes/no or safe/unsafe may not capture enough variation and nuanced perspectives (5F.6). It is therefore preferrable to avoid using safety questions with dichotomous response options (5F.6). It is also important to note that GBV risk and perceptions of safety are not fixed characteristics, and they can fluctuate due to various factors as well as from one person to another. There is limited guidance on how best to frame the safety related questions, the recall period to use and how to capture risks at different times of the day or related to different aspects of programming. When measuring safety in a given humanitarian context, it may be useful to try to identify and differentiate GBV risks that can be mitigated through program design and those that might not. This may include risks that were caused or exacerbated by the emergency or risks that are associated with or exacerbated by the humanitarian program itself (5F.7). This can help to identify which types of mitigation interventions should be prioritized. Use of secondary data and collaboration with GBV and protection colleagues can facilitate understanding of these risks.
6. Data Analysis and Use of Findings
  Even when data related to GBV risk mitigation are collected, M&E staff often are not trained on how to analyze that data, and program staff often do not know how to translate findings into program modifications or other actions. Key informants therefore indicated that M&E staff should be trained to analyze the M&E data collected (6.1), including analyzing proxy indicators for GBV risk, interpreting GBV risk findings within M&E data and using them to inform program adaptations and design of future programs. This will enable staff to address the important issues or risks related to the program immediately and adjust the intervention if needed. As part of this training, staff could be provided with examples of how they can adapt their programs or services based on specific findings.
  In general, M&E data should be regularly analyzed to assess progress towards targets (6.2). Staff should not only aim to assess the success of the program, but also measure program and data quality. For example, data quality monitoring can be facilitated with electronic surveys, which enable supervisors to measure how long each enumerator took to complete each survey and determine if they spent sufficient time explaining questions to participants. Analysis should include triangulation of data from multiple sources to assess consistency and the strength of the findings (6.3), and findings should be analyzed and reported with disaggregation by age, sex and disability status (6.4). Disaggregation of data was one of the most mentioned recommendations both within the literature and from the key informants. Monitoring data are key for day-to-day decision-making and to identify issues which require immediate action (6.5). M&E data could also help determine whether more intensive assessments in specific locations or on certain topics are needed.
7. Safety Concerns for Affected Populations and Staff
  The importance of considering the safety of both affected populations and humanitarian staff while designing and implementing GBV risk mitigation and M&E activities was emphasized across most documents and interviews. Staff members should know how to support survivors who willingly disclose their experience of GBV, including how to safely and ethically link them to services using a GBV referral pathway (7.1). Feedback mechanisms should be in place to monitor any negative consequences of GBV risk mitigation and M&E activities for affected populations and staff (7.4). Such mechanisms must take into account the needs of non-literate populations as well as minority languages and communication-related disabilities.
  Other promising practices include conducting *ongoing* risk analysis (7.2) to understand how activities may cause or exacerbate harm to affected populations and including a gender and GBV analysis as part of risk assessments (7.3). Conduct community safety mapping, which may include mapping of risks identified in safety audits (7.5). This can help to provide a holistic picture of the specific geographical locations or service delivery points that present potential risks and inform any restructuring or redesign of activities or site locations to reduce risks. To ensure the safety of staff and participants, staff should not complete forms in unsafe areas (7.6), and should stop data collection if at any point they determine that safety is at risk. Organizations have a duty of care to ensure that female staff have support while they are in the field (e.g., safety and security plans should be in place).
  Regarding the safety of affected populations during M&E activities, much of the guidance relates to the specific types of questions that staff ask during data collection, as well as following best practices for informed consent, confidentiality, responding to GBV disclosures, safe referrals and human subjects research, much of which is addressed in the data collection section. Data should be stored in accordance with data security protocols (paper documents are locked and electronic documents are password protected/encrypted) and only shared with relevant staff on a “need to know” basis (7.7).
8. Staff Capacity and Engagement
  Promising practices related to staff capacity and engagement were frequently highlighted across the literature and within the in-depth interviews and focus group discussions. For example, providing gender and GBV training for staff members working on risk mitigation activities (8.1) was noted as important to build foundational knowledge on GBV terminology and definitions and ensure consistency across key stakeholders, as well as strengthen knowledge and skills related to safety, referrals and doing no harm. Continual engagement with staff on gender and GBV, rather than one-time training, was underlined as important to deconstruct the biases of staff members themselves, and to track understanding and application of the information. It was also noted that when undertaking staff capacity strengthening activities, in addition to assessing completion rates, it is helpful to also measure change in attitudes and behaviors (8.7).
  Collective training, promoting exchange and dialogue and building trust between team members were noted to help ensure staff buy-in, a key element to undertaking effective M&E for GBV risk mitigation activities. Multiple sources describe how crucial staff buy-in is for both GBV risk mitigation programming and M&E. This process should begin well before the introduction of new tools or methodologies. Staff must be fully trained on the theory of change (when it exists) and program objectives, potential risks of conducting M&E of GBV risk mitigation and other related topics (8.2). This promising practice was one of the most common points mentioned across the literature and the from the key informants. One promising practice for achieving this degree of buy-in is to foster trusting relationships between GBV specialists and non-GBV teams and to engage in co-design of programs and M&E tools.
  One promising practice that was frequently highlighted in the reviewed documents and by key informants is that staff collecting data and involved in M&E should be trained on psychological first aid, ensuring confidentiality, informed consent, referring to GBV services and use of the GBV Pocket Guide (8.3). This ensures that in the event survivors do disclose to these non-GBV specialist staff they are properly prepared to support them without causing further harm. It is crucial that survivors have confidential avenues for reporting, and staff such as enumerators, who are directly engaging with the affected populations, should be trained on how to support survivors who willingly disclose their experiences. This includes referring to GBV response services, where they are available, and what to do in locations where such services are lacking. In these settings, the GBV Pocket Guide is the key resource to guide non-GBV specialists to support survivors and it uses a psychological first aid framework.
  M&E staff should receive training on how to analyze GBV risk mitigation data and how to translate the findings into program changes (8.4). Non-M&E staff should also receive guidance on how to adapt programming based on M&E findings. GBV specialists should conduct training (8.5) for non-specialist staff on relevant M&E tools. The growing need for GBV specialists who can strengthen capacity on GBV risk mitigation and accompanying M&E for colleagues in other sectors was highlighted. Several sources highlighted devoting more financial and human resources to recruitment of GBV specialists. Table 10 provides a case example on supportive supervision.

**Table 10:** Case example: supportive supervision to strengthen staff capacity on M&E

*Country*: Nigeria *Sector*: CCCM GBV specialists in Nigeria took a four-pronged approach to help strengthen the capacity of CCCM colleagues on systematic approaches to monitor and evaluate GBV risk mitigation activities. First, they held a series of workshops on GBV in certain sites to establish a more systematic way of identifying risks and determining potential actions to address the risks. Next, they trained staff on how to carry out observational safety audits at the sites to collect information on GBV-related risks. Third, the GBV specialists distributed guidance notes and provided supportive supervision on analyzing the data, especially in understanding how different proxy indicators might indicate risk and what the follow-up analysis should entail. For example, instead of asking community members directly if they feel safe accessing services, they may ask which areas people frequent most, and which they avoid. Finally, they worked with partners to develop action plans to address some of the problems they found with a focus on adapting their programs to further mitigate GBV risk. Furthermore, they had staff continue to monitor these program changes through standard post-distribution monitoring
*Key take away*: Engage GBV specialists to carry out capacity strengthening activities and supportive supervision with staff working in other sectors so that they have the skills to gather data on GBV risk, analyze it, and use it to inform program changes
  In addition, it was noted that self-care is an important and often overlooked issue. As staff, including M&E staff, may experience secondary trauma when discussing sensitive issues such as GBV risk, staff self-care should be addressed in training, self-care resources should be provided to staff and organizations should have clear staff care policies and offerings for staff to see trained counselors (8.6). Finally, it was emphasized that gender parity should be ensured among staff, particularly when activities involve direct contact with women and children (8.8), and the workplace should be a safe and supportive environment for female staff. Ensuring gender parity is particularly relevant for M&E staff, such as enumerators, as data collection can involve direct interaction with women, and same-sex interviews are a key promising practice.

## Discussion

This study identified a total of 92 current promising practices across 8 different categories relevant for measuring both protection and non-protection outcomes related to the implementation of GBV risk mitigation activities. We provide descriptions of each promising practice to help guide humanitarian operations, as well as the source(s) of the promising practice. All promising practices included directly apply to the M&E of GBV risk mitigation in humanitarian settings. However, a number of the promising practices for measurement may be broadly applicable to overall M&E activities, as well as both inside and outside of the humanitarian sector. In keeping with the original aim of the research, the process for determining inclusion of each promising practice attempted to align with current global guidance on GBV risk mitigation, such as the IASC GBV Guidelines; however, some recommendations from both the IDIs/FGDS and the desk review went beyond the scope of the Guidelines, in part reflecting the continually changing nature of this field [11]. The findings of this research are available as a resource for global and local humanitarian practitioners and researchers in diverse settings and sectors. The promising practices are supplemented with seven diverse case examples from various sectors that aim to illustrate the application of the promising practices using real-world examples. The case examples are intended to increase the understanding of this work and serve as valuable learning aids during capacity strengthening efforts. The promising practices and case examples are meant to complement and be utilized in conjunction with other GBV risk mitigation resources, namely the GBV Guidelines, GBV Minimum Standards, GBV Pocket Guide (including the mobile application version), and sectors’ own resources on GBV risk mitigation [11, 32, 33].

The term “best practices” is commonly used to denote approaches that, through rigorous analysis, have been identified as the most successful at achieving a specific aim. In this paper, we intentionally chose to use the term “promising practices” to emphasize the remaining need for more rigorous methodological research on these practices, as well as evaluation of GBV risk mitigation activities and monitoring approaches. The impetus for this research comes directly from the dearth of such rigorous evaluation of GBV risk mitigation and any subsequent evidence-based findings. The combination of a systematic desk review, IDIs and FGDs enabled us to gather a large number of perspectives to determine currently employed promising practices and allows for an important level of detail and nuance to the analysis. However, we cannot make statistically significant claims about our findings. Provision of the source(s) of the promising practices provides insights into and contextualization of the identified promising practices but should be interpreted with caution. The study was designed to capture a broad range of promising practices but not to prioritize, rank or compare the degree of consensus across each of the promising practice. Further validation, testing and prioritization could be an avenue for future research and discourse.

Some of the promising practices identified in this study seemed to have broad consensus behind their utility, but no clear agreement on the actual means of carrying them out. For example, many of the reviewed documents and key informants mentioned that questions framed around women and girls’ perceptions of safety are generally more appropriate than asking about personal experiences with GBV. However, neither the existing literature nor key informants presented a standard and validated approach or methodology for measuring safety perceptions, or a prescribed set of circumstances or conditions that must be present to safely assess these perceptions. Further research is needed to strengthen the evidence base on these promising practices and address key emerging questions about delivering safe and effective humanitarian programming. This includes methodological research to advance the GBV risk mitigation field toward “gold standard” measurement approaches that are valid, reliable and accurate, as well as research to demonstrate effectiveness of GBV risk mitigation strategies with respect to both GBV and sector-specific outcomes.

While the research for this study was conducted in 2019 and prior to the Coronavirus Disease 2019 (COVID-19) pandemic, the landscape of the humanitarian sector has since shifted in an effort to reduce spread of COVID-19 and limit deaths and adverse health effects among displaced populations. Many standard humanitarian interventions, norms, and so-called best practices have been redesigned and adapted to fit a new reality of limited in-person interaction and stay at home orders, including in displacement settlements and other humanitarian settings. With emerging evidence about how COVID-19 response measures have exacerbated GBV risk globally [39–41], GBV risk mitigation and the promising practices identified in this document are more relevant than ever and should be adopted and put into practice by all humanitarian practitioners. However, several aspects of the promising practices may need to be modified during the time of COVID-19 and potentially permanently moving forward as the humanitarian sector adopts precautions for any future outbreaks. In fact, adaptability is highlighted as a promising practice itself, including in 2A.11 and 2B.3. Many organizations and researchers—including authors of documents reviewed or those interviewed—presented recommendations for modifying interventions to COVID-19, and the GBV Guidelines implementation team has additionally released guidance on GBV risk mitigation within the context of COVID-19 together with a compilation of related resources [42]. Practitioners should take into account these recommendations in addition to the promising practices identified above.

In general, the promising practices identified should always be considered within the specific context in which a humanitarian actor is placed. While the identified promising practices were included specifically because there was a broader level of agreement about their merit, there were at times differences in opinion between members of different humanitarian sectors or those operating in different types of humanitarian contexts. Thus, any approaches to GBV risk mitigation outlined above should be adapted to the given context and, when possible, piloted before being formally introduced into humanitarian programs and operations.

The desk review, IDIs and FGDs all pointed to differences in the extent to which various humanitarian sectors engage with M&E of GBV risk mitigation. Some sectors—such as the WASH sector—have invested heavily in GBV risk mitigation and monitoring of these efforts. The crucial role that individuals in specific leadership positions have played in prioritizing these efforts within a given sector or country operation was highlighted as one contributing factor to this heterogeneity. The differences across sectors may present an opportunity for sectors to learn from those that have more experience, and ultimately signals that more work needs to be done to secure buy-in and develop field-friendly methods and tools that are relevant and appropriate for each sector as they seek to increase their GBV risk mitigation efforts. Additional work is also needed at the inter-sectoral level (multi-sector needs assessments, monitoring and reporting templates, etc.) and with senior management to firmly embed safety considerations with women and girls into the core of humanitarian response operations. Furthermore, potential misunderstandings and/or tensions that can arise between non-protection specialists and protection or GBV actors as they coordinate to develop humanitarian programs and determine priorities in program design were described as a barrier to adoption of GBV risk mitigation and its M&E. While all key informants confirmed their commitment to GBV risk mitigation activities and recognized their importance, some also described the difficulty that non-protection actors can have in adapting their standard activities and M&E approaches to include new approaches for GBV risk mitigation. These reflections illustrate the need for more and continued coordination between GBV and non-GBV actors to easily facilitate uptake of GBV risk mitigation promising practices so that non-GBV actors can adapt them in a way that works best for their sector.

### Limitations

It should be noted that these promising practices are driven to some degree by the available literature, including published and grey literature in formal reports and documents. Some field knowledge and experiences are not captured in such documents, reports and articles, and qualitative data collection was therefore conducted to ensure field lessons and insights on the measurement of GBV risk mitigation were included. However, it is possible that some important perspectives and relevant lessons were not included. In particular, due to limitations in the purposive sampling design and difficulty connecting with field-based staff, there are gaps in representation of national staff and local organizations and some geographic regions in our sample. The research was also confined by language restrictions as interviews and reviewed documents were in English. Furthermore, the desk review in particular focused mostly on documentation from the Protection, WASH, Shelter, CCCM and Livelihoods areas and these sectors were also better represented among the key informants.

Finally, the level of available evidence supporting the different categories of promising practices was variable. In some areas, such as monitoring outputs or outcomes of GBV risk mitigation activities especially around safety or how to integrate this type of measurement into existing M&E frameworks, there was limited information available. This is a reflection of the fact that M&E of GBV risk mitigation is a particularly novel area of programming and research. This paper synthesizes and presents the current state of knowledge on the measurement of GBV risk mitigation.

## Conclusion

In summary, this paper provides a broad list of promising practices to strengthen the measurement of both GBV and non-GBV outcomes related to the implementation of GBV risk mitigation activities in humanitarian response. This list represents a foundation upon which further research and analysis can be built to validate specific practices and generate in-depth guidance for their implementation. Case examples are also presented to illustrate real-world applicability of several of these practices. The promising practices and case examples are designed to complement and be utilized in conjunction with other available GBV risk mitigation resources.

## Data Availability

Survey materials are available by request to the corresponding author.

## Abbreviations

CCCM: Camp coordination and camp management
COVID-19: Coronavirus Disease 2019
DRC: Democratic Republic of the Congo
FGD: Focus group discussion
GBV: Gender-based violence
HHI: Harvard humanitarian initiative
IASC: InterAgency Standing Committee
IDI: In-depth Interview
IHRL: International Human Rights Law
IRB: Institutional Review Board
IRL: International Refugee Law
IPV: Intimate partner violence
M&E: Monitoring and evaluation
WASH: Water, sanitation and hygiene
WFP: World Food Program
